# Hepatitis B virus prevalence and risk factors in hard-to-reach Turkish population living in Belgium: A protocol for screening

**DOI:** 10.1097/MD.0000000000015412

**Published:** 2019-05-03

**Authors:** Özgür M. Koc, Niel Hens, Rob Bielen, Pierre Van Damme, Geert Robaeys

**Affiliations:** a Department of Gastroenterology and Hepatology, Ziekenhuis Oost-Limburg, Genk; b Faculty of Medicine and Life Sciences, Hasselt University, Hasselt, Belgium; c Department of Medical Microbiology, School of Nutrition and Translational Research in Metabolism, Maastricht University Medical Centre, Maastricht, the Netherlands; d Interuniversity Institute for Biostatistics and Statistical Bioinformatics (I-Biostat), Hasselt University, Hasselt; e Centre for Health Economic Research and Modelling Infectious Diseases, Vaccine and Infectious Disease Institute, Antwerp University; f Centre for the Evaluation of Vaccination, Vaccine and Infectious Disease Institute, Antwerp University, Antwerp; g Department of Gastroenterology and Hepatology, KU Leuven, Leuven, Belgium.

**Keywords:** Belgium, hepatitis B, migrant, prevalence, screening, Turkish

## Abstract

**Background:**

Hepatitis B virus (HBV) infection is an important public health problem in the Turkish population, that is, one of the largest migrant populations in Europe. With the introduction of cost-effective antiviral treatments in the past decade, there is a need to identify HBV-infected patients who may benefit from treatment. This study describes the design of a study to assess the HBV prevalence in the Turkish population living in Belgium. Additionally, we will determine the risk factors of HBV infection and the uptake of screening, vaccination, and antiviral treatment in this hard-to-reach Turkish population.

**Methods:**

A longitudinal, epidemiological study will be conducted in the region Middle Limburg Belgium, where the Turkish adult population, 18 years of age and older, will be screened for hepatitis B surface antigen (HBsAg), antibodies against HBsAg (anti-HBs), and antibodies against hepatitis B core antigen (anti-HBc). Educational meetings concerning viral hepatitis B will be organized and there will be 3 ways to be screened for HBV: immediately after the educational meetings, at the Outpatient Hepatology Department of Ziekenhuis Oost-Limburg, and at home visits. Subsequently, participants will be asked to fill in a questionnaire regarding sociodemographic factors, migration history, risk factors for HBV infection (e.g., sharing toothbrushes, HBV-infected family member), and HBV vaccination status. Six months after screening, HBsAg-positive patients will be assessed whether they are under follow-up at the general practitioner or hepatologist. We will also gather information regarding the uptake of vaccination in nonimmunized subjects.

**Discussion:**

This study will provide information about the HBV prevalence and distribution of the stages of liver disease in the Turkish population in Belgium. By determining the risk factors for HBV infection, subgroups with an increased prevalence of HBV infection can be identified.

**Clinical trial number:**

This clinical trial is registered at clinicaltrials.gov (NCT03396458).

## 1 Introduction

Hepatitis B virus (HBV) infection is an important public health problem. In the year 2017, approximately 257 million people are chronically infected worldwide and at risk of developing serious sequelae, such as cirrhosis and hepatocellular carcinoma.^[1,2]^

According to the WHO, Turkey is one of the countries with intermediate endemicity for hepatitis B. The hepatitis B surface antigen (HBsAg) prevalence thereby changes from 2% to 3% in the Western part of the country to 7% to 8% in Eastern part.^[3]^ In intermediate endemic countries, such as Turkey, horizontal HBV transmission by nonsexual close contact besides perinatal, sexual, and parenteral transmission is the main route of infection.^[4–7]^ Risk factors associated with horizontal transmission in childhood are poor socioeconomic conditions, the presence of a HBsAg-positive family member and the habit of sharing various personal and household articles within the home (e.g., contaminated toothbrush, contaminated towels).^[5,8,9]^

In contrast, the HBsAg prevalence is low (<1%) in the general population of Belgium and many other Western European countries.^[10,11]^ Sexual transmission of HBV in persons with high-risk sexual behavior (e.g., men who sex with men, heterosexual persons with multiple sex partners) remains a common source of HBV transmission in these low endemic countries.^[2]^

Starting in 1961, political, economic, and social developments led Turkish people to migrate to Europe, Australia, Arab countries, and former Soviet Union countries.^[12]^ According to Turkish government statistics, 5.5 million Turkish citizens live abroad, of whom around 4.6 million live in Western European countries.^[13]^ Turkish citizens are therefore one of the largest migrant groups in Europe. Moreover, a recent study by Ahmad et al^[14]^ indicated that the Turkish population was ranked 3rd as migrant group with the highest absolute number of chronic HBV cases in Europe. However, their estimates of HBsAg prevalence were based on prevalence studies conducted in Turkey. Another study conducted by a Dutch group showed that the Turkish population in the Netherlands had a low level of awareness and knowledge regarding HBV infection which could subsequently impinge the attitude toward HBV screening.^[15]^

Nonetheless, little is known about the true prevalence and risk factors of HBV infection among the Turkish population living in Western European countries. Considering the fact that infection with HBV in children most often result in chronic infection^[16]^ and universal HBV vaccination in Belgium and Turkey only started after, respectively, 1999^[17]^ and 1998^[3]^, an appreciable number of the Turkish population in Belgium may be infected with HBV. Many of the HBV-infected Turkish patients may also remain undiagnosed, since the onset of HBV and progression to cirrhosis and hepatocellular carcinoma is generally asymptomatic and occur many years after exposure.^[18]^ As HBV treatment options have greatly improved over the past decades and have proven to be cost-effective, there is a need to identify chronic carriers who may benefit from treatment.^[19–21]^ Therefore, this study aims to assess the prevalence and risk factors of HBV infection in hard-to-reach Turkish population living in Belgium. The identification and early antiviral treatment of HBV infection in the Turkish population could prevent the development of end-stage liver disease and further spread, while the associated health cost burden in this group at risk can be reduced.

## 2 Methods/design

### 2.1 Study design

The current study is a longitudinal, epidemiological study.

### 2.2 Study population

The study population consists of Turkish individuals, living in the region Middle Limburg in Belgium.

Inclusion criteria:

- Eighteen years and older of age
- Turkish individuals (defined as persons who are born in Turkey or persons of whom one of the parents is born in Turkey)
- All participants irrespective of being aware of having been tested and knowing their HBV status
- All participants irrespective of being aware of having been fully vaccinated
- Signed informed consent form

Minors, incapacitated subjects, and non-Turkish individuals were to be excluded from the study.

### 2.3 Pre-screening phase

In the pre-screening phase, we developed a brochure, flyer, poster, and video focusing on HBV infection. The patient information and consent form, questionnaire, brochure, flyer, poster, and video were translated in the Turkish language by a certified translator. The final information in Dutch and Turkish language were then thoroughly checked by delegates of Turkish associations and the questionnaire was pilot tested by 10 persons of Turkish origin, first and 2nd-generation migrants, males and females, from different age categories (age group 18–39 years, 40–59, 60–79, and 80+).

During the study, participants will be able to contact the first author by phone or e-mail for Turkish/Dutch information and questions. This study will be communicated to the association of general practitioners (GPs) in Genk, a city in the region Middle Limburg, who will be kept well-informed throughout the study.

### 2.4 Recruitment

An estimated 17,510 Turkish persons live in the region Middle Limburg, of whom approximately 11,540 are 18 years of age and older (Limburg in cijfers, personal communication). Before the start of the screening phase, focus group discussions were held with delegates of 4 mosques in Middle Limburg (Waterschei, Winterslag, Kolderbos and Sledderlo) and 2 Turkish responsibles of the Genk city council. Group discussions included general information on hepatitis B (e.g., hepatitis B occurs more often in Turkey compared to Belgium, routes of transmission, asymptomatic nature of disease, disease outcome), prevention (e.g., screening, vaccination), design of this study, recruitment, and conduct of the study. Being familiar with the stigma regarding hepatitis in the Turkish community, several motivating factors and barriers to go for HBV screening were identified during the group discussions. Tables 1 and 2 illustrate the motivating factors and barriers by social-cultural, religious, and other factors as well as the appropriate actions in the current screening study.

**Table 1:**
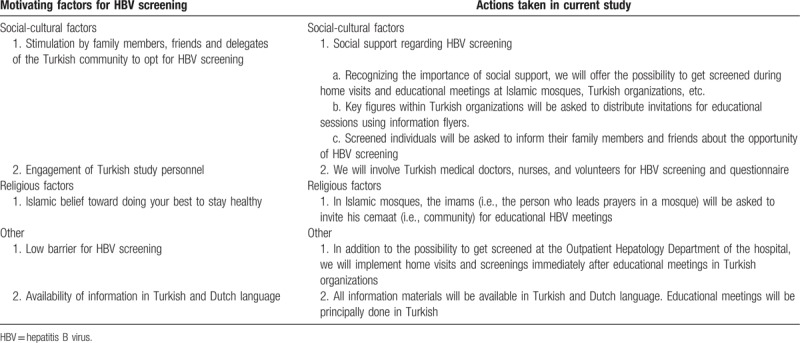
Motivating factors for hepatitis B virus screening identified during focus group discussions and actions taken in current study.

**Table 2:**
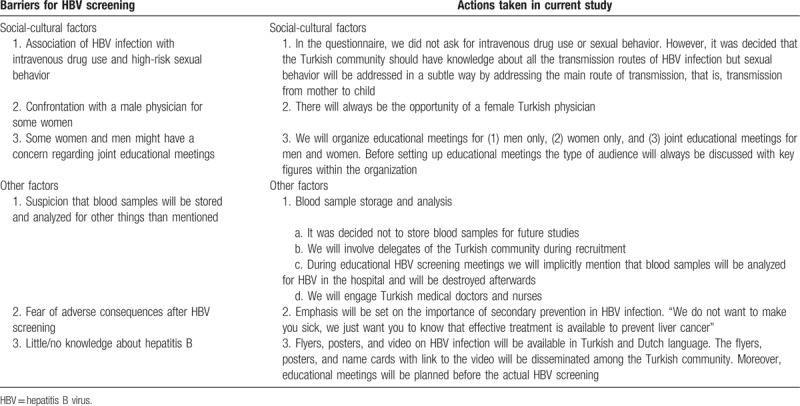
Barriers for hepatitis B virus screening identified during focus group discussions and actions taken in current study.

As the Islamic mosques mainly consist of male members/visitors, we will also search for female Turkish organizations in the Middle Limburg area. From September 2017 until August 2019, with the support of key figures within these organizations, we will organize educational meetings with the possibility for immediate HBV screening afterwards. The educational and screening sessions will be scheduled after the regular meetings of each organization.

**Figure 1:**
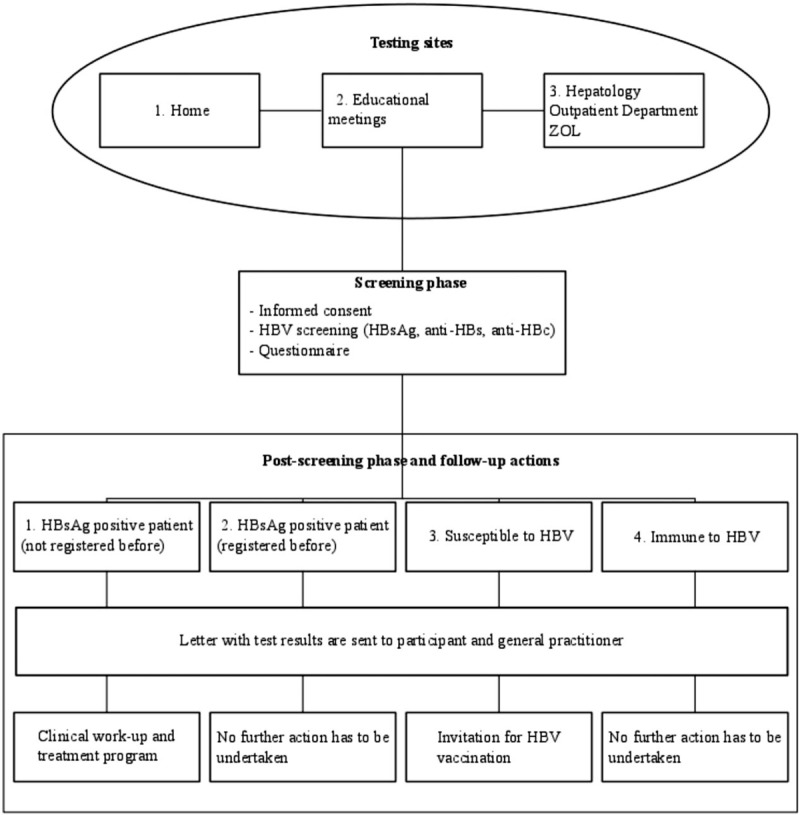
Flowchart of the study design. HBV = hepatitis B virus, HBsAg = hepatitis B surface antigen, anti-HBs = antibodies against hepatitis B surface antigen, anti-HBc = antibodies against hepatitis B core antigen, ZOL = Ziekenhuis Oost-Limburg.

The key figures within these organizations will distribute invitations for the sessions using information flyers, posters, and name cards with a link to the hepatitis B video. The flyers, posters, and video contain information about risk factors for horizontal transmission (sharing personal and household articles, such as toothbrushes) and vertical transmission in addition to general information such as HBV vaccination, consequences of unrecognized HBV, and the possibility of antiviral treatment. Turkish persons who are unable to attend a session will be invited to visit additional educational meetings and screening sessions in Ziekenhuis Oost-Limburg (ZOL) or can make an appointment with Ö.M.K at the Hepatology Outpatient Department (OPD) of ZOL. In addition to the abovementioned methods, Turkish people can also contact the first author for home visits with the possibility to sign an informed consent and be screened for HBV. Figure 1 shows the flowchart of the study design.

### 2.5 Screening phase

The educational sessions will be held in Turkish by the first author who will address the risk factors, routes of transmission, the fact that screening is free of charge, consequences, prevention, and HBV treatment. After the educational meetings, there will be the possibility to be screened for HBV. Those who opt for screening will be given an informed consent form and a questionnaire, available in Dutch and Turkish. HBV screening will also be possible at home visits and at the OPD of ZOL. Blood specimens will be transported to and processed in the laboratory of ZOL.

### 2.6 Laboratory testing

All blood samples will be tested for HBsAg, antibodies against HBsAg (anti-HBs), and antibodies against hepatitis B core antigen (anti-HBc) using an electrochemiluminescence assay (Cobas 8000 e602, Roche, Germany). The interpretation of positive and negative results will be carried out as recommended by the test producer.

### 2.7 Post-screening phase and follow-up actions

Patients who test positive for HBsAg will have the opportunity to enter an OPD. HBV screening results will be sent by letter to the patients and to their GP. A clinical work-up and treatment program in the OPD will be proposed for newly diagnosed patients. No further action will be undertaken in previously registered HBsAg-positive patients.

Nonimmunized subjects and their GP will be informed about the test results by a letter. Nonimmunized subjects will additionally be asked to consult their GP for HBV vaccination. In case of immunization against HBV infection, only a letter regarding the HBV screening results will be sent to the participant as well as to the GP.

### 2.8 Sample size

Sample size calculation was performed with the aid of Epi Info (version 7.2, Centers for Disease Control and Prevention [CDC], Atlanta, GA). Considering the lack of data on the number of first and second-generation Turkish migrants living in Middle Limburg, the number of samples per age was calculated instead so that the results of the sample group agree with that of the Turkish population in Middle Limburg with a confidence interval of 95% and 3% as an acceptable margin of error. The expected frequencies for the different age categories 18 to 39, 40 to 59, 60 to 79, and 80+ are 54%, 35%, 9%, and 1%, respectively (Limburg in cijfers, personal communication). Our analysis would lead to a total of 1000 tested persons.

### 2.9 Measures

**Figure 2:**
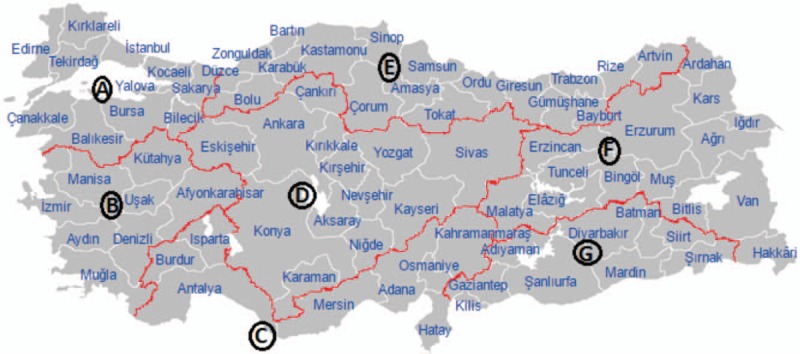
Map of Turkey according to the geographical regions. Map of Turkey according to regions: (A) Marmara, (B) Aegean, (C) Mediterranean, (D) Central Anatolia, (E) Black Sea, (F) Eastern Anatolia, and (G) Southeastern Anatolia.

Questionnaire: we searched PubMed to identify articles on risk factors for HBV infection in the Turkish community using the following terms and keywords alone and/or in appropriate combinations: hepatitis B, Turkey, Turkish, Turks, risk factor. No publication date or language restrictions were set. In addition, reference lists of all identified articles were checked to identify additional material, including (grey) literature. The final questionnaire includes the following risk factors: blood transfusion (no, yes before 1974 in Turkey, yes before 1974 in Belgium, other), dental treatment (yes in Turkey, other), (if female) gynecological examination (no, yes in Turkey, yes in Belgium, other), (if male) circumcision (no/yes), (if male) circumcision carried out by a doctor (no, yes, unknown), (if male) way of circumcision (alone, collective, unknown), surgery (no, yes in Turkey, yes in Belgium, other), treatment with needles (no, yes in Turkey, yes in Belgium, other), HBV-infected mother (no/yes), HBV-infected father (no/yes), HBV-infected brother (no/yes), HBV-infected sister (no/yes), HBV-infected partner (no/yes), other infected family member (no/yes), sharing toothbrushes with other household members (no, yes once, yes on regular basis), sharing nail clippers with other household members (no/yes), sharing razors with other household members (no/yes), sharing used towels with other household members (no/yes), eating from the same plate as other household members (no/yes), tattoo/piercing/earlobe perforation (no, yes in Turkey, yes in Belgium, other), treatment with Fish spa (no, yes in Turkey, yes in another country than Turkey). Furthermore, the questionnaire will collect data on HBV vaccination (vaccinated no/yes/unknown), (if yes) number of vaccinations, (if no) reasons of not being vaccinated) and previous HBV test results. Data regarding sociodemographics (age, gender, educational status of mother, educational status of father), first language (Dutch/Turkish), migration history (country of birth, (if born in Turkey) year of migration to Belgium, mother's country of birth, father's country of birth, (if mother born in Turkey) region of birth from mother, (if father born in Turkey) region of birth from father) will be collected to characterize the study population. Regarding region of birth, Turkey will be divided in the 7 geographical regions: Marmara, Aegean, Mediterranean, Central Anatolia, Black Sea, Eastern Anatolia, and Southeastern Anatolia (Fig. 2).^[3]^ The questionnaire will not cover questions regarding intravenous drug use or sexual behavior as these questions could lead to a significant lower participation rate and the answers to these questions are not reliable in this Turkish population with a majority being Muslim.

Clinical outcomes: within 6 months of a HBsAg-positive test result, we will assess whether or not HBsAg-positive patients are under follow-up at the GP or hepatologist. Clinical follow-up data will be collected: coinfection (hepatitis C virus, hepatitis delta virus, HIV); determination of liver disease stage (via fibroscan, ultrasonography abdomen, magnetic resonance imaging of the liver, duodenoscopy, and, if necessary, liver biopsy); and treatment eligibility, initiation and outcomes. Outcomes of Fibroscan and/or liver biopsy will be scored according to the METAVIR classification system for fibrosis (F0: no fibrosis, F1: portal fibrosis without septa, F2: few septa, F3: numerous septa without cirrhosis, F4: cirrhosis).^[22]^ Within 6 months after HBV screening, we will also gather data regarding nonimmunized subjects, namely, if they initiated HBV vaccination by the GP.

### 2.10 Primary outcome measure

The primary outcome variable is the prevalence of current infection with HBV in the Turkish population living in Middle Limburg. For this reason, one Li-heparin tube collection and subsequent HBsAg analysis will be performed from each participant.

### 2.11 Secondary/exploratory outcome measures

The questionnaire will provide information on secondary outcomes by assessing the risk factors for recent or past HBV infection (anti-HBc positivity). We will also determine the willingness for HBV assessment in the Turkish population in Middle Limburg. This will be assessed by the number of persons who did go for HBV testing divided by the total population who attended one of the educational meetings, the OPD of ZOL or home visit. Other secondary outcome measures are percentage of new HBsAg-positive patients that are under follow-up at the GP or a hepatologist, factors associated with HBsAg-positive persons to be under follow-up at the GP/hepatologist 6 months after HBV screening test result, distribution of stages of liver disease in HBsAg-positive patients, treatment eligibility, initiation, and outcomes in HBsAg-positive patients, the percentage of Turkish population who are immune against HBV infection based on anti-HBs status, and percentage of Turkish population who are susceptible for HBV infection, and uptake of HBV vaccination in nonimmunized Turkish subjects.

### 2.12 Statistical analyses

Survey data will be entered into a secure electronic database Castor EDC (Castor Electronic Data Capture, Ciwit Bv, Amsterdam, the Netherlands). Coded data analyses will be performed using SPSS (Release 25, Armonk, NY). Categorical data will be analyzed with the chi-squared test or Fisher's exact test. Differences in 2 and several continuous variables will be assessed by the independent *t* test and one way-ANOVA test, respectively. The normality and homogeneity of variances will be verified, respectively, by Shapiro-Wilk and Levene values >0.05. In case the assumptions for parametric tests are violated, the equivalent Mann–Whitney test and Kruskal-Wallis test will be applied for comparing 2 and several continuous variables, respectively. A multivariate logistic regression model will be used to identify risk factors associated with anti-HBc total positivity. Risk factors that are found to be significantly associated (*P* < .05) with anti-HBc total positivity in univariate analysis will be included as covariates in the logistic regression model. Since all included samples will be linked to questionnaires, corrections for over or undersampling of certain groups will be performed. In order to do this poststratification, weights for age and gender will be created. This will be done for the 2 variables separately, and for their interaction. Chi-squared tests will then be performed again, this time including the poststratification weights. The same will be done for logistic regression. The level of statistical significance is set at *P* < .05.

### 2.13 Ethical approval and informed consent

The Ethical Committee of ZOL Genk and Hasselt University approved this study with protocol version 1.0 dated July 13, 2017 (B371201732623) and written informed consent will be obtained from all study participants.

### 2.14 Availability of data and materials

The datasets used during the current study will be available from the corresponding author on reasonable request.

## 3 Discussion

Immigration to Western Europe has a long history, but increased at an unprecedented rate over the past decades. During this time period, the incidence and mortality of hepatocellular carcinoma increased in Western Europe, likely in part due to the increased migration from countries with intermediate (HBsAg prevalence 2–7%) or high (>8%) HBV prevalence.^[14,23]^

Moreover, screening in migrants from countries with an intermediate HBV endemicity, such as the Turkish population in the Netherlands and Germany, has shown a 15 to 25 times higher HBsAg prevalence of 3% to 5% in comparison to the general population.^[24,25]^ However, data on the real HBV prevalence in the Turkish population residing in Belgium and data on the risk factors and distribution of stages of liver disease in this Turkish population are not available. Nonetheless, knowledge on the HBV prevalence, risk factors, and clinical evolution in Western Europe is necessary.

### 3.1 Strengths and limitations

This study will determine the prevalence and risk factors of HBV infection in a large panel of first and 2nd-generation Turkish migrants living in Middle Limburg, Belgium. It will show whether the HBV prevalence obtained from this Turkish population study is higher than the HBV prevalence found in previous studies conducted in the general population living in Belgium. The inclusion of large number 2nd-generation migrants will also provide information whether 2nd-generation Turkish migrants are at risk for HBV infection in addition to 1st-generation migrants. In the Netherlands, a study on 103 2nd-generation Turkish migrants could not find a difference in HBsAg and anti-HBc seroprevalence from the native population.^[24]^ Considering the small sample size of the Dutch study, in view of variations in HBV prevention and control strategies between European countries, it is important to assess the issue of HBV infection in the 2nd-generation migrants living in Belgium together with 1st-generation migrants.^[26]^

In contrast to cross-sectional studies, this longitudinal follow-up study will be able to differentiate between acute/chronic HBV cases and false-positive HBsAg test results. It will also provide information on linkage to care and stages of liver disease which may assist physicians, public health practitioners, and policymakers in eliminating hepatitis B. The assessment of anti-HBs in addition to HBsAg and anti-HBc will give us insight in the proportion of individuals susceptible to HBV infection. Furthermore, this study will gather information on risk factors for HBV infection, sociodemographics, migration history, and clinical data (e.g., HBsAg positivity). Combined, this information could help identify how HBV infection in this Turkish population is spreading so that further spread can be stopped. This study will also provide information on the feasibility and acceptability of HBV screening in hard-to-reach migrant groups.

There are some limitations to the present study. First, this study could be underpowered to find significant associations between certain risk factors and HBsAg or anti-HBc, as sample size calculation was performed on distribution of age categories and not on expected HBsAg or anti-HBc prevalence. Second, a concern is that, even though risk factors for HBV infection were recorded, certain risk behaviors could have been underreported due to social desirability bias. Third, it is unknown whether observations from Turkish population in Middle Limburg could be used as an estimate for Europe. However, collection of demographic factors allows subanalyses. Fourth, the questionnaire will not cover points regarding intravenous drug use or high-risk sexual behavior as these questions could lead to a significant lower or biased participation rate and the answers to these questions are ought to be unreliable in this Turkish population in which the majority of people identify with Islam.

## Author contributions

**Conceptualization:** Özgür M Koc, Niel Hens, Rob Bielen, Pierre Van Damme, Geert Robaeys.

**Data curation:** Özgür M Koc, Geert Robaeys.

**Formal analysis:** Özgür M Koc, Niel Hens.

**Funding acquisition:** Özgür M Koc, Geert Robaeys.

**Investigation:** Özgür M Koc.

**Methodology:** Özgür M Koc, Niel Hens, Rob Bielen, Pierre Van Damme, Geert Robaeys.

**Project administration:** Özgür M Koc, Geert Robaeys.

**Resources:** Özgür M Koc, Geert Robaeys.

**Supervision:** Niel Hens, Pierre Van Damme, Geert Robaeys.

**Writing – original draft:** Özgür M Koc.

**Writing – review & editing:** Niel Hens, Rob Bielen, Pierre Van Damme, Geert Robaeys.

Özgür M Koc orcid: 0000-0003-3678-5703.
